# Deoxyribonuclease 1-like 3 may be a potential prognostic biomarker associated with immune infiltration in colon cancer

**DOI:** 10.18632/aging.203173

**Published:** 2021-06-22

**Authors:** Jing Liu, Jingya Yi, Zhihong Zhang, Donglin Cao, Lei Li, Yachao Yao

**Affiliations:** 1 Department of Laboratory Medicine, Guangdong Second Provincial General Hospital, Guangzhou 510317, China; 2 The Second School of Clinical Medicine, Southern Medical University, Guangzhou 510317, China; 3 Center for Reproductive Medicine, The Third Affiliated Hospital of Guangzhou Medical University, Guangzhou 510150, China; 4 Key Laboratory for Reproductive Medicine of Guangdong Province, Guangzhou 510150, China

**Keywords:** colon adenocarcinoma, deoxyribonuclease 1-like 3, tumor microenvironment, immune infiltration, The Cancer Genome Atlas (TCGA) Database

## Abstract

Colon adenocarcinoma (COAD) is a common cancer of the digestive system. It’s high morbidity and mortality make it one of the leading causes of cancer deaths. In this study, we studied the microenvironment of colon cancer to find new diagnostic markers and immunotherapy targets for colon cancer. Tumor purity of colon cancer samples in TCGA database were obtained by ESTIMATE algorithm. Then, we analyzed the association of Immune, Stromal, and Estimate scores with tumor prognosis and clinicopathological features. By comparing the gene expression profiles between tumor and normal samples, the high and low immune score groups, 117 intersecting differentially expressed genes (DEGs) were obtained. The function, molecular pathway, and prognostic value of these 117 DEGs pointed toward the importance of deoxyribonuclease 1-like 3 (DNASE1L3). Validation results from multiple databases showed low expression of DNASE1L3 in colon cancer. A single GSEA and correlation analysis of immune cells indicated that DNASE1L3 was closely related to immunity. The low expression of DNASE1L3 in colon cancer samples was measured with qRT-PCR. The scratch and cell proliferation experiments suggested that DNASE1L3 may affect cell migration. Therefore, we concluded that DNASE1L3 might be a biomarker associated with prognosis and immune infiltration in colon cancer.

## INTRODUCTION

Colon adenocarcinoma (COAD) is a malignant tumor occurring in the colon. The specific etiology of COAD is unclear and may be associated with the patient’s weight, poor eating habits, environment, and heredity, among other factors [1, 2]. Currently, the main treatment for COAD is surgery combined with chemoradiotherapy; however, the curative effect is poor, and the postoperative quality of life is seriously affected for stage III and IV patients due to relapse and the high incidence of complications after surgery [3]. At present, most colon cancer patients are diagnosed at an advanced stage, and the incidence of the disease is gradually showing a trend towards development in younger patients [4]. Therefore, it is crucial to strengthen the early diagnosis and treatment of COAD and accelerate research of the mechanism of this disease.

The tumor microenvironment (TME) refers to the tumor cells and their environment, and mainly includes tumor cells, immune cells, fibroblasts, stromal cells, and their secretion products (such as cytokines and chemokines). The immune and matrix components are the main elements of the non-tumor components in the TME. The dynamic balance between tumor cells and their microenvironment is the basis of tumor cell growth, invasion, and drug resistance [5, 6]. With the recent exploration of the TME, a growing number of studies have been devoted to the exploration of drugs targeting the TME, providing new ideas for tumor therapy.

The Cancer Genome Atlas (TCGA) database is the largest cancer gene information database, which can help analyze genetic changes in the process of tumorigenesis and development, thus improving cancer prevention, diagnosis, and treatment [7]. In this study, we determined the presence of the biomarker deoxyribonuclease 1-like 3 (DNASE1L3) by analyzing the transcriptome data and microenvironment of COAD in the TCGA database. DNASE1L3, also known as deoxyribonuclease γ, is a member of the deoxyribonuclease 1 family. It is secreted by macrophages and dendritic cells and can cleave single- or double-stranded DNA with DNA hydrolysis activity [8]. However, the role and prognostic value of DNASE1L3 in colon cancer has not been reported. For this reason, our study regarding the association between DNASE1L3 and COAD may contribute to the understanding of the pathogenesis of colon cancer and may provide a theoretical basis for the clinical search for effective therapeutic targets for colon cancer.

## RESULTS

### Correlation analysis of survival and clinicopathological characteristics

**Figure 1:**
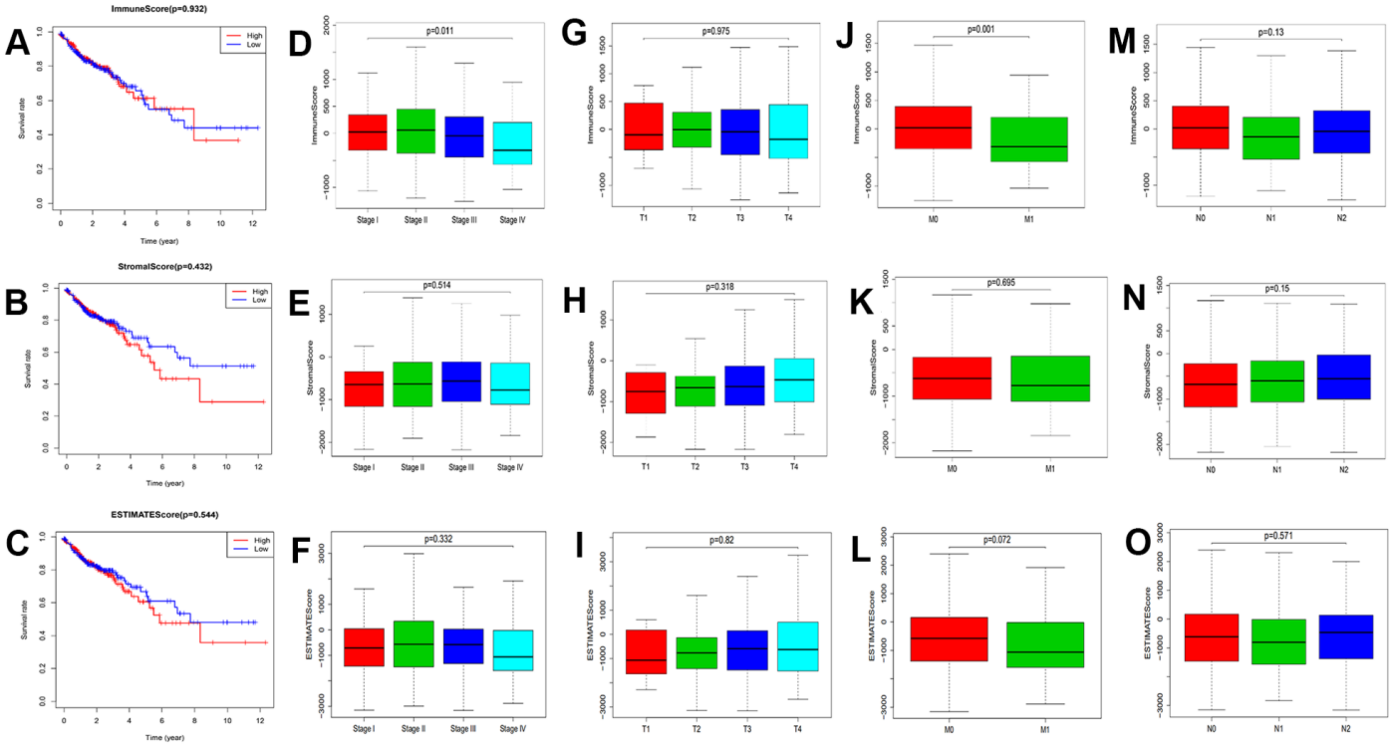
**Correlation between prognosis and clinicopathological characteristics.** (**A**–**C**) Correlation analysis between scores and survival of colon cancer patients (*p*=0.932, 0.432, and 0.544 for ImmuneScore, StromalScore, and ESTIMATEScore, respectively; log-rank test). (**D**–**F**) Correlation analysis of ImmuneScore, StromalScore, and ESTIMATEScore with stage (*p*=0.011, 0.514, and 0.332, separately; Kruskal–Wallis rank sum test). (**G**–**I**) Correlation analysis of three scores with T classification (*p*=0.975, 0.318, and 0.82, respectively; Kruskal–Wallis rank sum test). (**J**–**L**) Correlation analysis of ImmuneScore, StromalScore, and ESTIMATEScore with M classification (*p*=0.001, 0.695, and 0.072, separately; Wilcoxon rank sum test). (**M**–**O**) Correlation analysis of Immune, Stromal, and ESTIMATE Scores with N classification (*p*=0.13, 0.15, and 0.571, respectively; Kruskal–Wallis rank sum test).

Survival analysis was conducted to assess the correlation between TME components and the survival rate in colon cancer patients (Figure 1A–1C). There was no statistically significant difference among the overall survival of these three scores (*p*>0.05). Next, we analyzed the correlation between the clinicopathological characteristics of the patients and scores (Figure 1D–1O). There were significant differences between the Immune Score and M classification in the tumor–node–metastasis (TNM) Classification of colon cancer (Figure 1J). At the same time, the immune score was also related to the stage of patients (Figure 1D). However, the clinical characteristics was no significant correlation with Stromal and Estimate score. These results suggested that the immune components of the TME may be associated with the progression of colon cancer. Therefore, we selected the Immune Score for subsequent analysis.

### DEGs screening and function analysis

**Figure 2:**
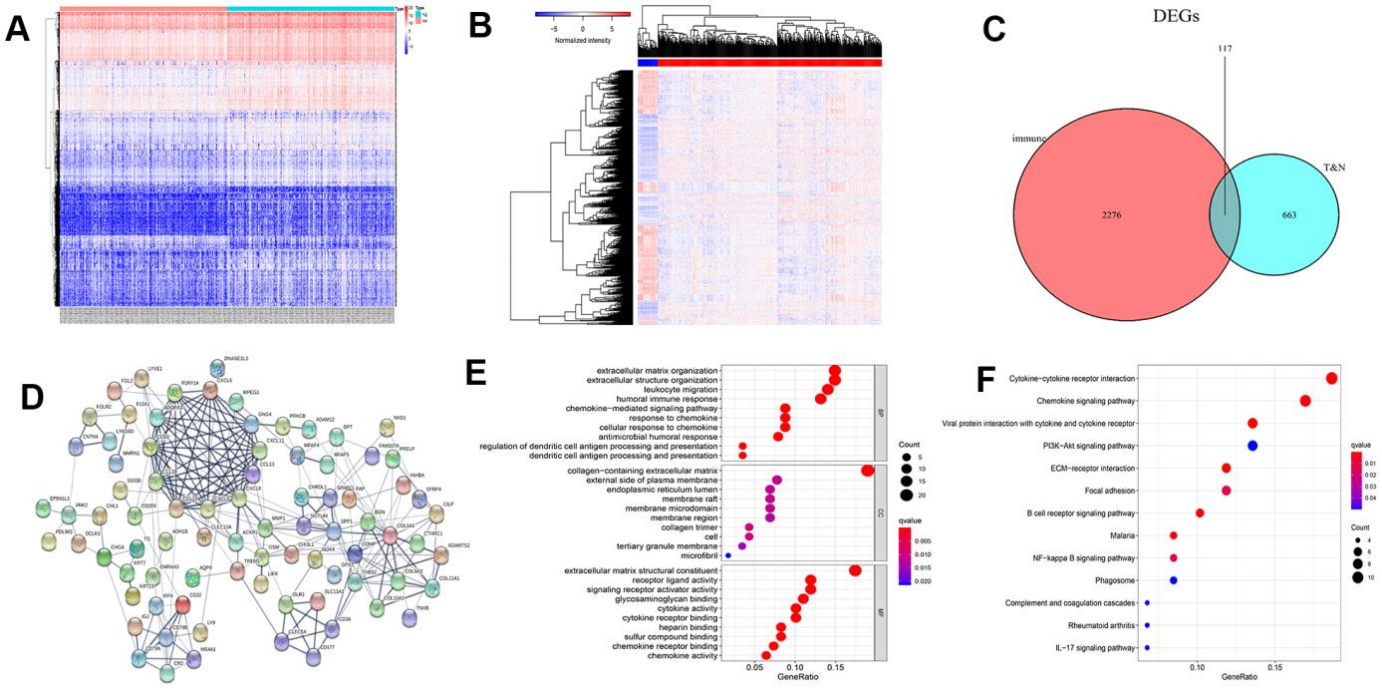
**Heatmap of differentially expressed genes (DEGs), Venn diagram, protein-protein interaction (PPI) network, and enrichment analysis.** (**A**) Heat map of immune-related DEGs. (**B**) Heatmap of DEGs between colon cancer and normal samples. (**C**) Venn diagram of genes associated with immunity in normal and cancer samples. (**D**) PPI network of 82 core DEGs. Gene Ontology (**E**) and Kyoto Encyclopedia of Genes and Genomes enrichment (**F**) analysis for 117 DEGs.

By comparing and analyzing the gene expression profiles of the two groups (low vs. high Immune Score), we obtained 2,393 differentially expressed mRNAs (DEmRNAs). The heatmap of these DEmRNAs is presented in Figure 2A. Similarly, we analyzed the DEGs in colon cancer (480 cases) and normal samples (41 cases), and obtained 780 DEmRNAs. The heatmap is presented in Figure 2B. Then, we intersected the immune score-related DEmRNAs with the DEmRNAs in colon cancer and normal samples, and got 117 intersecting DEGs (Figure 2C).

To explore interactions between proteins of DEGs, we put these 117 genes into the STRING database. By using the “hide disconnected nodes in the network” condition, a protein-protein interaction (PPI) network of 82 core genes was demonstrated (Figure 2D). The results of Gene Ontology (GO) functional enrichment analysis demonstrated that these genes were primarily enriched in the extracellular matrix, humoral immune response, and leukocyte migration (Figure 2E). Kyoto Encyclopedia of Genes and Genomes (KEGG) enrichment analysis demonstrated that these genes were mainly involved in cytokine receptors and chemokine signaling pathway (Figure 2F).

### Survival analysis of DEGs

**Figure 3:**
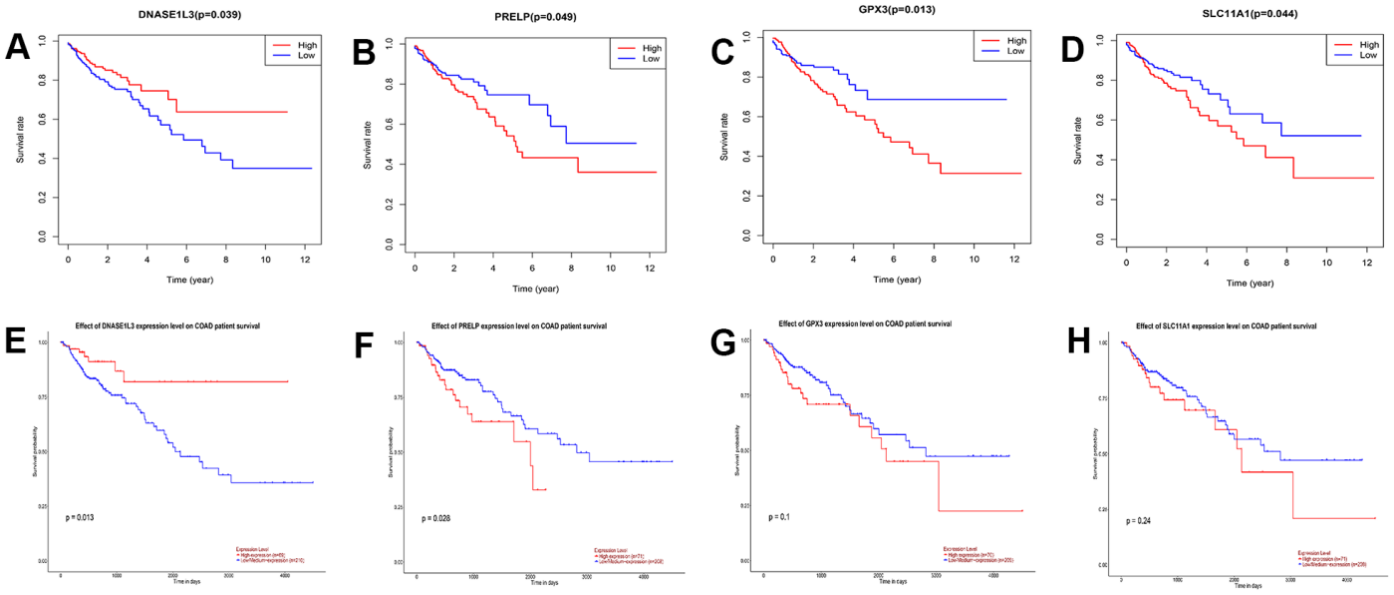
**Survival analysis of differentially expressed genes (DEGs) in The Cancer Genome Atlas and validation in UALCAN database.** (**A**–**D**) Survival curves of genes with *p*<0.05 in 117DEGs, log-rank test. (**E**–**H**) Survival analysis of DNASE1L3, PRELP, GPX3, and SLC11A1 was verified using the UALCAN database (*p*=0.013, 0.028, 0.1, and 0.24, respectively; log-rank test).

The survival and survminer R packages were used to perform survival analysis and draw the survival curve for the 117 DEGs. Four genes associated with prognosis were obtained: *GPX3, DNASE1L3, SLC11A1,* and *PRELP* (Figure 3A–3D). We verified these four genes on the UALCAN database for survival analysis, and found that two genes, *DNASE1L3* and *PRELP*, were indeed significant for the prognosis of colon cancer patients (Figure 3E–3H). Based on a search of the literature, we found that the oncogenic role of *PRELP* in colon cancer has been previously reported. Therefore, the gene *DNASE1L3* was selected for follow-up analysis.

### DNASE1L3 expression of colon cancer and its correlation with clinicopathological parameters in TCGA

**Figure 4:**
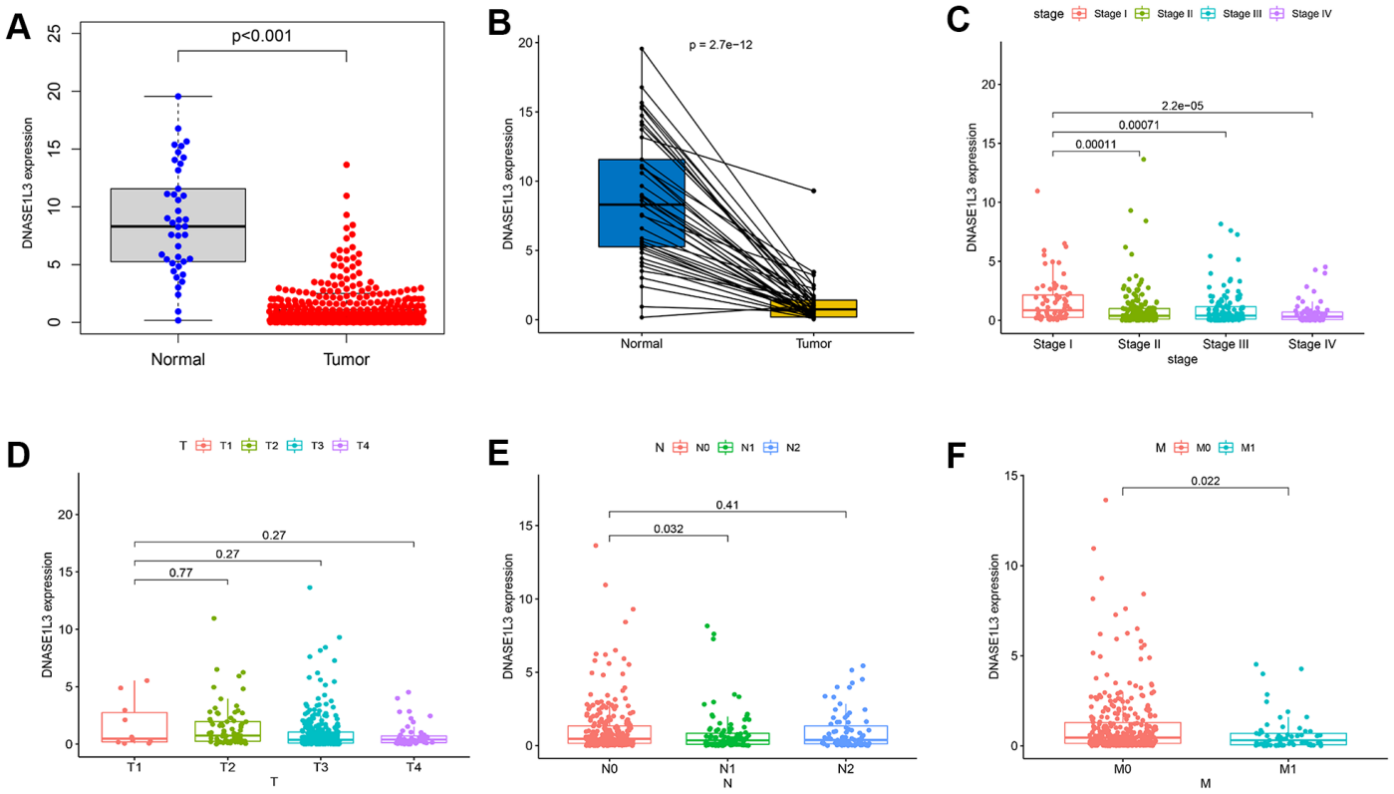
**Expression of deoxyribonuclease 1-like 3 (DNASE1L3) in colon cancer and its correlation with clinicopathology.** (**A**) The expression of DNASE1L3 in colon cancer and normal samples (*p*<0.001, Wilcoxon rank sum test). (**B**) Expression analysis of DNASE1L3 in colon cancer and paired normal samples (*p*=2.7e-12, Wilcoxon rank sum test). (**C**–**F**) The association between DNASE1L3 and clinicopathology (Kruskal–Wallis rank sum test or Wilcoxon rank sum test, statistical significance at *p*<0.05).

In the Cancer Genome Atlas (TCGA) database, we compared DNASE1L3 expression of colon cancer samples with normal samples at the mRNA level and found that DNASE1L3 expression was significantly lower in the tumor group than in the normal group (Figure 4A). The paired analysis showed the same results (Figure 4B). Next, we analyzed the association between DNASE1L3 and clinicopathology (Figure 4C–4F), and found that the expression of DNASE1L3 was related to stage and M classification (distant metastasis) of patients (*p*<0.05). These results suggest that *DNASE1L3* may be a tumor suppressor gene associated with prognosis in colon cancer.

### Verification of DNASE1L3 expression levels in multiple databases

**Figure 5:**
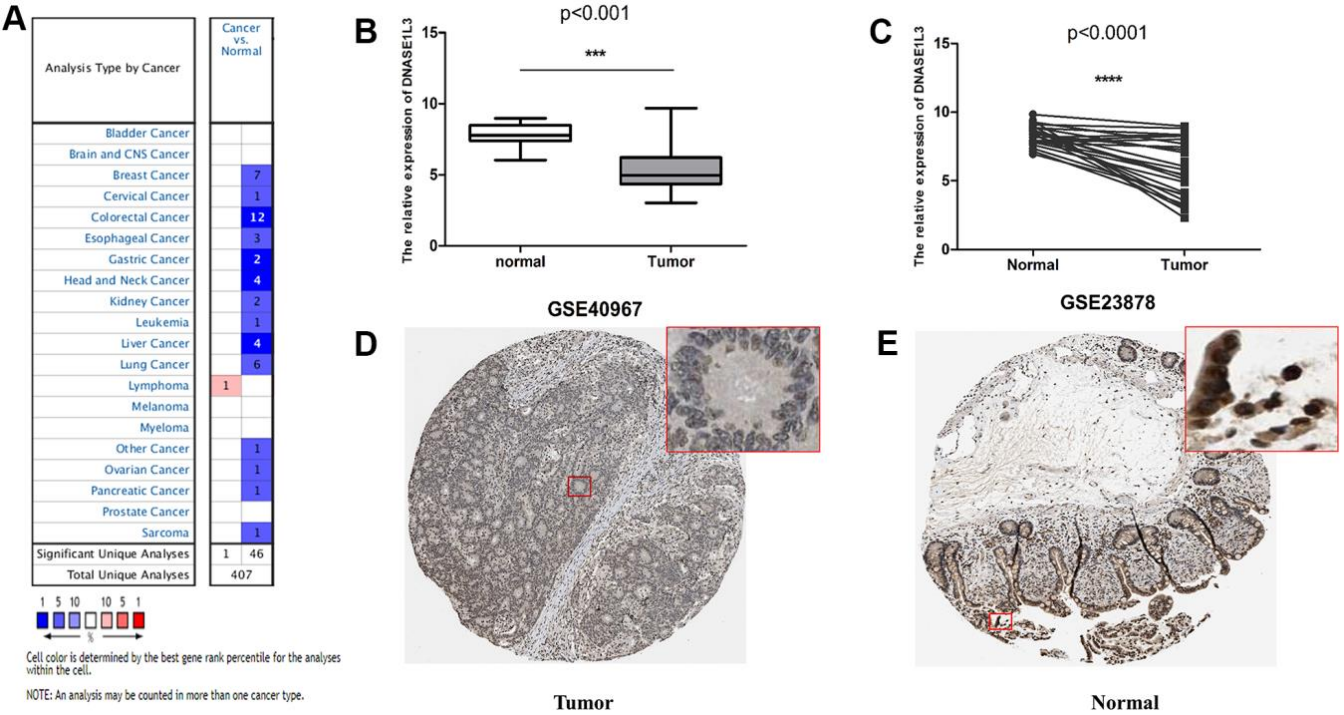
**Verification of the expression level of deoxyribonuclease 1-like 3 (DNASE1L3) using various databases.** (**A**) DNASE1L3 pan-cancer analysis using the Oncomine database. (**B**) Differential expression of DNASE1L3 in GSE40967. ***, *p*<0.001, Wilcoxon rank sum test. (**C**) The matching analysis of DNASE1L3 in GSE40967. ****, *p*<0.0001, Wilcoxon rank sum test. Immunohistochemical results of DNASE1L3 in colon cancer (**D**) and normal intestinal mucosa (**E**) in The Human Protein Atlas (THPA) database, antibody: Sigma-Aldrich HPA019955, rabbit., location: nuclear.

To further validate DNASE1L3 expression, we analyzed it in multiple databases. First, the Oncomine database was used to investigate the mRNA expression of DNASE1L3 in multiple different tumors. In cancer samples such as colorectal cancer, gastric cancer, head and neck cancer, and liver cancer, DNASE1L3 was significantly downregulated compared with normal tissues (Figure 5A). Gene Expression Omnibus (GEO) validation cohorts (GSE40967 and GSE23878) also showed a significant decrease in DNASE1L3 expression in colon cancer compared with normal tissue (Figure 5B, 5C). DNASE1L3 protein expression levels from The Human Protein Atlas (THPA) database obtained the same conclusion (Figure 5D, 5E).

### Single gene set enrichment analysis

**Figure 6:**
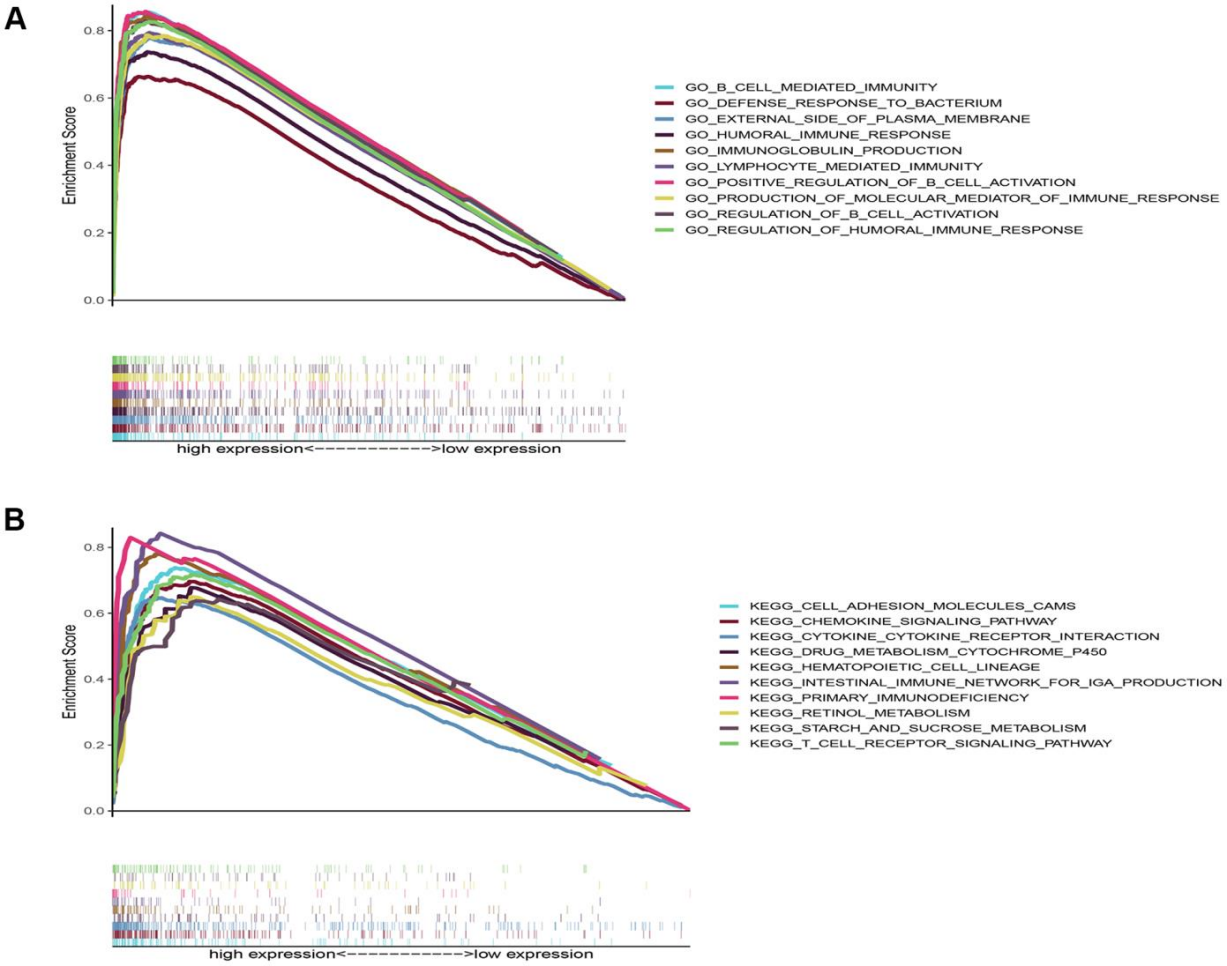
**Gene set enrichment analysis results of deoxyribonuclease 1-like 3’ s high expression.** (**A**) The top 10 results from the Gene Ontology analysis. (**B**) The top 10 results from the Kyoto Encyclopedia of Genes and Genomes analysis. *P* value <0.05 and false discovery rate <0.05.

To explore the potential biological functions and pathways of DNASE1L3 in COAD, we performed gene set enrichment analysis (GSEA) based on DNASE1L3 mRNA expression. Because the results of the DNASE1L3 low-expression group showed p values >0.05, we only visualized the KEGG and GO analysis results of the high-expression group (Figure 6). The top 10 GO analyses demonstrated that genes in the DNASE1L3 high-expression group were mainly enriched in immune-related activities, such as immunoglobulin production, immune response, and B cell- and lymphocyte-associated immunological activities. Immune-related pathways in the top 10 pathways from the KEGG analysis were “immune network for immunoglobulin A production” and “primary immune deficiency.” These results indicated that DNASE1L3 was highly correlated with immunity.

### Correlation analysis between DNASE1L3 and immune cells

**Figure 7:**
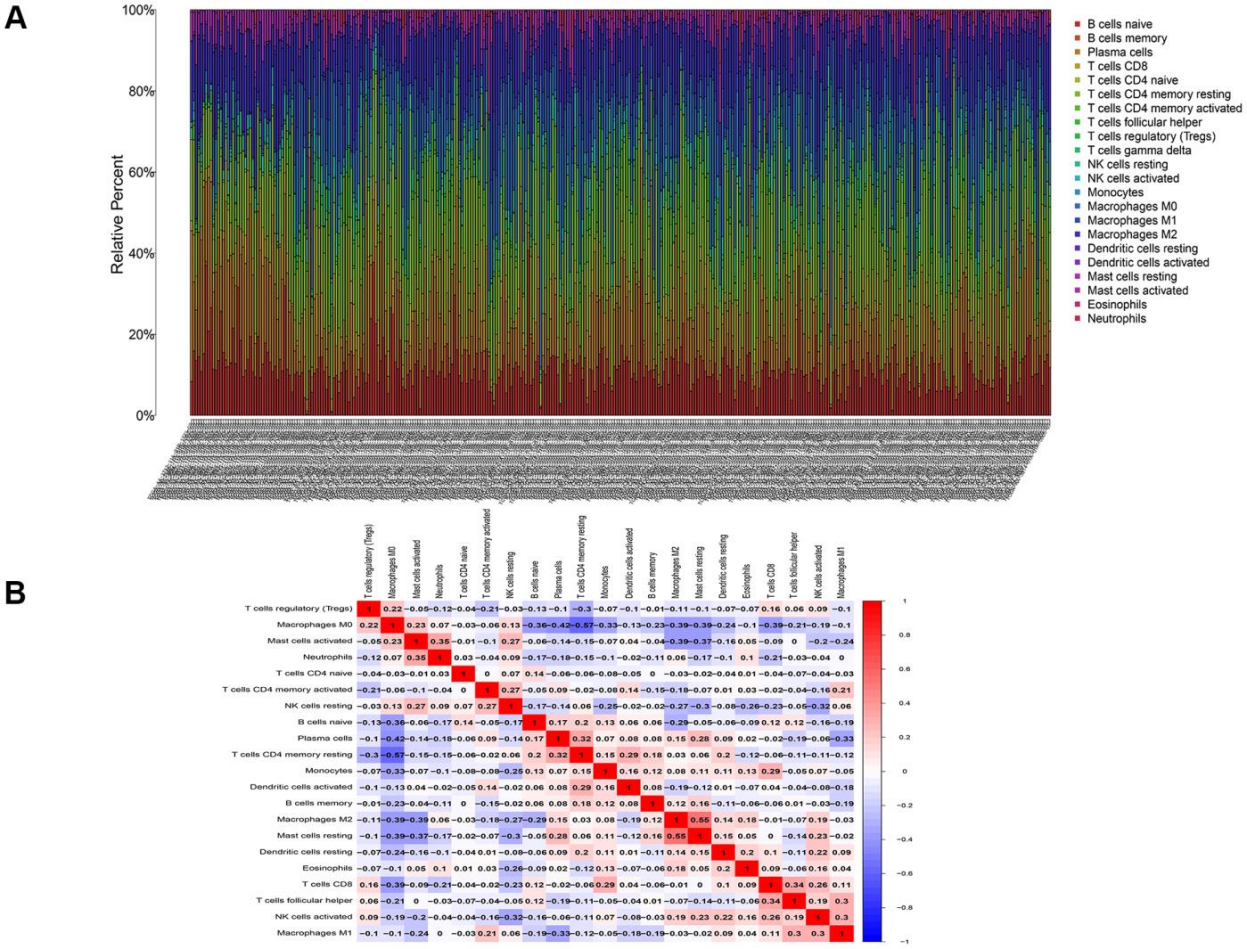
**Infiltration of immune cells in colon cancer.** (**A**) The proportion of immune cells in each colon cancer sample. (**B**) Co-expression of immune cells in colon cancer.

**Figure 8:**
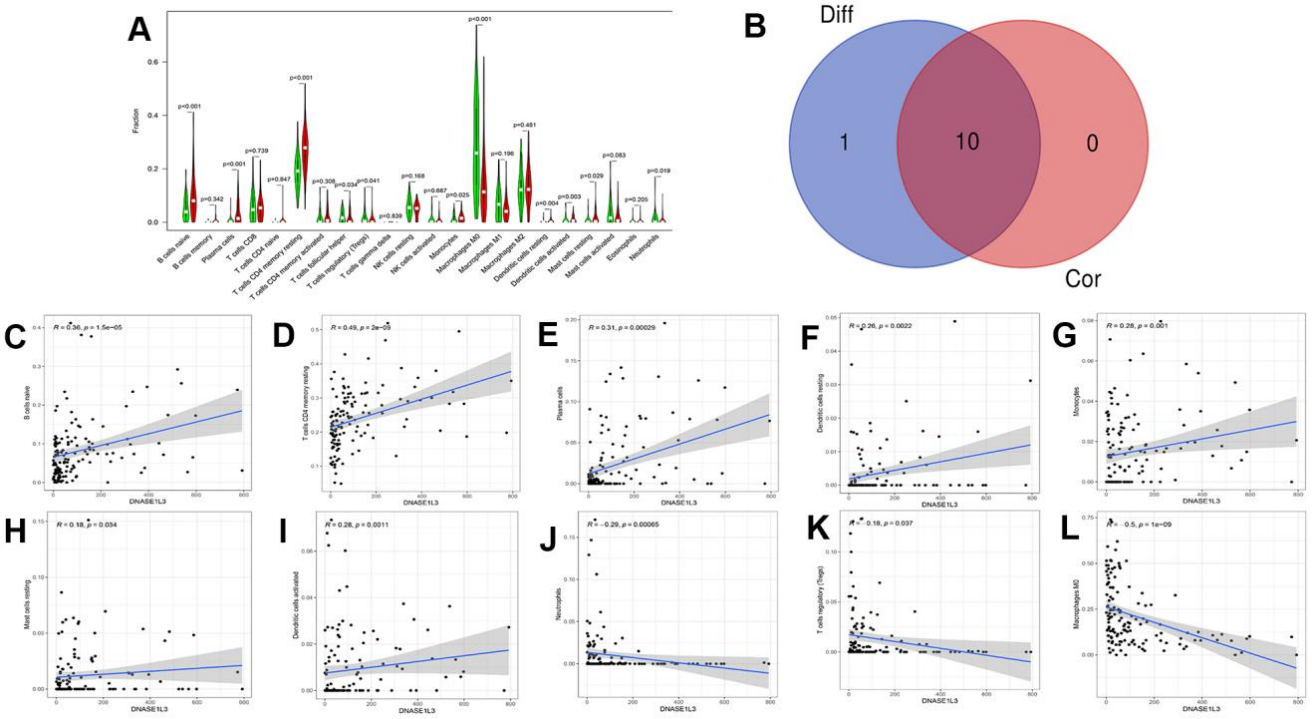
**Immune cells associated with high and low deoxyribonuclease 1-like 3 (DNASE1L3) expression levels.** (**A**) Immune cells related to DNASE1L3 expression were obtained through differential expression analysis (statistical significance was set to *p*<0.05). (**C**–**L**) 10 kinds of immune cells related to DNASE1L3 were obtained by the method of correlation test (*p*<0.05). (**B**) A Venn diagram of intersecting immune cells types between differential expression analysis and correlation tests.

To analyze the correlation between DNASE1L3 and immune cells in colon cancer, we used the CIBERSORT algorithm to determine the content of immune cells in each colon cancer sample. A heatmap of the proportion of immune cell types in each sample and the co-expression of immune cells are presented in Figure 7. Based on the correlation test and the differential expression analysis, we obtained a total of 10 immune cells associated with DNASE1L3 (Figure 8 and Supplementary Table 1). Among them, seven immune cell types were positively correlated with DNASE1L3 expression level (R>0 and p<0.05). The remaining three immune cell types were negatively correlated with DNASE1L3 expression level (R>0 and p<0.05). In addition, we also found that macrophage M0 was most correlated with DNASE1L3 (P=1.01, R=-0.5). These results further confirm that DNASE1L3 is significantly associated with immune infiltration in colon cancer.

### DNASE1L3 low expression in colon cancer tissue samples, and its exploration on the motor ability of proliferation and movement in colon cancer cells

**Figure 9:**
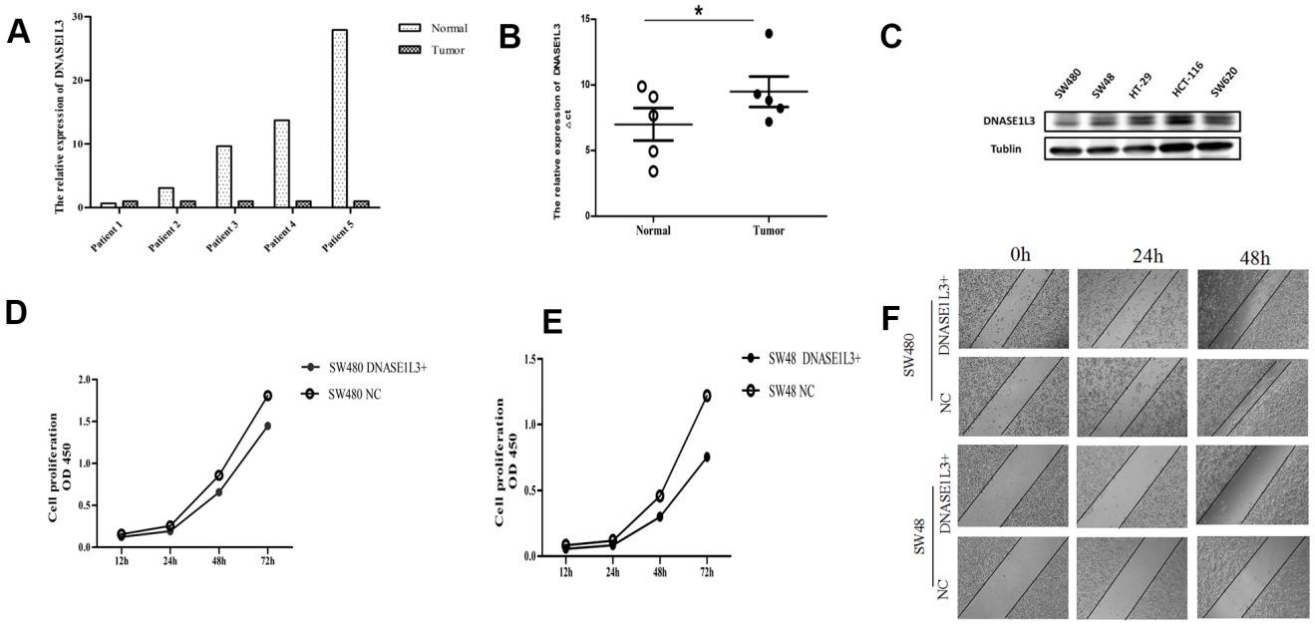
**Expression of DNASE1L3 in colon cancer tissue samples and its effect on proliferation and motor ability of colon cancer cells.** (**A**) The expression of DNASE1L3 in colon cancer and paracancerous tissue samples was detected by RealTime-PCR (2^-ΔΔCt^ was used as the calculation method, normalized with β-actin). (**B**) Comparison of ΔCt values of colon cancer and paracancerous tissue samples homogenized by internal reference β-actin (paired T test, * *P*=0.036). (**C**) The expression of DNASE1L3 in 5 colon cancer cell lines detected by Western blot. (**D**, **E**) The cell proliferation ability was detected by CCK8 in SW480 (**D**) and SW48 cells (**E**) after transfection with DNASE1L3+ plasmid or NC plasmid (*P*=0.75, 0.60, respectively). (**F**) Wound healing assay *in vitro* was used to evaluate the ability of wound closure in SW480 and SW48 cells after transfection with DNASE1L3+ plasmid or NC plasmid (1h, 24h, 48h photography, ×10).

To verify the accuracy of the above bioinformatics analysis, RT-qPCR was performed on the surgically resected colon cancer and its paired adjacent cancer samples (Figure 9A). The results showed that DNASE1L3 expression was lower in 4 out of 5 pairs of colon cancer tissue samples than in paracancerous tissues (4/5, 80%). Next, we analyzed the differential expression of DNASE1L3 based on ∆Ct values in 5 pairs of samples, and the results showed that there were indeed a difference in DNASE1L3 expression between the tumor group and the paracancerous group, and the difference was statistically significant (Figure 9B, P=0.036). These suggested that DNASE1L3 was under-expressed in colon cancer tissues. In order to explore the effect of DNASE1L3 on the function of colon cancer cells, the Western blot was used to detect the basic protein expression of DNASE1L3 in 5 colon cancer cell lines: SW480, SW48, HT-29, HCT-116 and SW620 (Figure 9C). Based on protein expression result, SW480 and SW48 cells with the lowest expression of DNASE1L3 among the 5 colon cancer cells were selected for subsequent cell function experiments. In the CCK8 cell proliferation experiment (Figure 9D, 9E), the cell proliferation ability of the SW480 and SW48 were slightly weaker than the control group after transfected with the DNASE1L3 overexpression plasmid, but there was no significant statistical difference between the two group. The scratch test results of two colon cancer cell lines showed that the scratches of the SW480 control group almost disappeared at 48h, while the difference between the scratches of the SW48 two groups was not obvious (Figure 9F), this meant that DNASE1L3 might regulate the healing ability of colon cancer cells *in vitro*.

## DISCUSSION

By analyzing the TME components of colon cancer in the TCGA database, we found that the expression of DNASE1L3 is decreased in colon cancer and it is associated with prognosis, disease progression, and immune invasion in colon cancer. A series of subsequent analyses and experiments confirmed these hypotheses.

The TME can inhibit or promote cancer [9]. For example, macrophages in the TME can promote the formation of tumor blood vessels, leading to distant invasion of tumor cells [10]. However, Treg cells play an anti-tumor immune role by reducing the immunity of tumor-associated antigen-specific T cells [11]. In contrast to both of these conditions, dendritic cells (DCs) induce T cellular responses and reduce cancer progression [12]. Therefore, the disorder of the microenvironment may be an important cause of cancer [13]. Comprehensive analysis of the role of TME in colon cancer has a profound significance in the diagnosis and treatment of colon cancer. Immunotherapy has been widely used in cancer therapy research in recent years and has made significant progress, including immune checkpoint blockade therapy, chimeric antigen receptor T cell therapy, oncolytic virus, and tumor-infiltrating lymphocyte therapy [14–17].

DNASE1L3 is associated with autoimmune diseases. It can digest chromatin in microparticles released by apoptotic cells, which is a potential autoantigen. Its excessive accumulation will cause the body to produce an autoimmune reaction and anti-DNA antibodies [18–20]. Neutrophil extracellular traps (NETs) are a special form of extracellular DNA deposition, and DNASE1L3 can cooperate with macrophages to mediate the degradation of NETs and promote NET clearance [21]. The absence or mutation of DNASE1L3 can cause the body to produce an anti-DNA reaction [22] leading to autoimmune diseases, such as systemic lupus erythematosus, rheumatoid arthritis, systemic sclerosis, autoimmune hemolytic anemia, and ankylosing spondylitis [23–25]. For instance, Sherry et al. found that eight genes, including *DNASE1L3,* could be used to distinguish early and late stages of renal clear cell carcinoma patients through multiple gene-based threshold models [26]. Furthermore, Wang et al. found that DNASE1L3 could be used as an independent prognostic factor for the survival of patients after radical resection of liver cancer [27]. One study suggested that DNASE1L3 deficiency may cause lymphoma cells to become resistant to VP-16 [28]. These findings suggest that DNASE1L3 is closely associated with tumors.

In this study, we found that DNASE1L3 had a negative correlation with the prognosis of colon cancer patients and was gradually downregulated with the progression of the disease. Combined with immune-cell infiltration analysis, we found that many immune cells in colon cancer were associated with DNASE1L3 expression. This suggests that DNASE1L3 may regulate colon cancer immunity by regulating multiple immune cell groups. For example, high DNASE1L3 levels can increase immune system toxicity by increasing infiltration of monocytes, plasma cells, DCs, resting mast cells, resting T cells, and naïve B cells, therefore activating the immune system and increasing immune toxicity in colon cancer patients. These results are consistent with previous reports on the protective effects of DNASE1L3.

In conclusion, this study reveals that DNASE1L3 may be an important biomarker associated with immune infiltration in colon cancer and will provide a theoretical basis for the selection of immunotherapy targets for colon cancer.

## MATERIALS AND METHODS

### Test datasets from the TCGA database and validation datasets from the GEO database

Test datasets of 521 colon cancer transcriptomes and patient clinical information were downloaded from the TCGA database (https://portal.gdc.cancer.gov/), including 480 colon cancer samples and 41 normal samples. Validation datasets of GSE40967 and GSE23878 were downloaded from the GEO database (https://www.ncbi.nlm.nih.gov/geo/). GSE40967 included 566 primary colorectal adenocarcinoma samples and 19 non-tumoral colorectal mucosa samples, and GSE23878 included 35 colon tumor samples and 24 normal paired tissue samples. These data were derived from TCGA and GEO, both of which are publicly available and open access; therefore, there was no requirement for ethics committee approval.

### Tumor purity analysis

We analyzed the tumor purity of the 480 COAD samples in the TCGA database using the ESTIMATE R package, and obtained the Immune Score, Stromal Score, and ESTIMATE Score of each of the samples. We sorted colon cancer samples according to these scores, and the samples were then divided into two groups according to the median of the three scores for subsequent correlation analysis.

### Survival and clinicopathological characteristics analysis

Detailed survival data were available for 454 of the 480 colon cancer patients; therefore, these 454 patients were included for subsequent survival and clinicopathological characteristics correlation analysis. The survival and survminer R packages were used to perform the overall survival analysis and to draw the survival curve. The Wilcoxon and Kruskal–Wallis rank sum tests were used to analyze the correlation between clinicopathological characteristics and scoring. Statistical significance was set at p<0.05.

### Differential expression analysis

The DESeq2 R package was used to analyze the DEmRNAs associated with the immune score of colon cancer (480 tumor samples, |log fold change|>1 and adjusted p value <0.05) and the DEmRNAs between 480 tumor samples and 41 normal samples (|log fold change|>2) and adjusted p value <0.05). After determining gene intersection, 117 DEGs associated with immunity in colon cancer and normal samples were obtained.

### Protein-protein interaction network

The PPI network for intersection of DEGs was constructed using the STRING database (https://string-db.org/), and the network diagram of these 81 genes was displayed by conditional network display options set to accommodate disconnected nodes in the network.

### Gene ontology and pathway enrichment analysis

To explore the potential functional and molecular pathways of DEGs, we performed GO and KEGG pathway enrichment analysis and visualization of 117 DEGs using the clusterProfiler R package. Statistical significance was set to p<0.05 for enrichment analysis.

### The UALCAN database

UALCAN database (http://ualcan.path.uab.edu/) is an online site for analyzing and mining cancer data, primarily based on cancer data in the TCGA databases [29]. In this study, we used this database for survival analysis validation.

### Gene set enrichment analysis

GSEA is a computational method used to determine whether a predefined set of genes can exhibit significant consistency in the differences in two biological states [30]. We divided COAD RNA-seq data into high-expression and low-expression groups according to DNASE1L3 expression, and GSEA_4.1.0 was used for analysis. The number of permutations was set to 1000. Statistically significant gene sets were considered with a p value <0.05 and false discovery rate <0.05.

### Oncomine database

The Oncomine database (https://www.oncomine.org/resource/main.html) is the world's largest oncogene chip database, with the most complete cancer mutation profile, gene expression data, and related clinical information [31]. In our study, this database was used to perform the DNASE1L3 pan-cancer analysis at the mRNA level.

### The human protein atlas

The THPA database (https://www.proteinatlas.org/) provided tissue and cell distribution information for 26,000 human proteins. This database comprises the Tissue Atlas, Cell Atlas, and Pathology Atlas [32]. We used the database to analyze DNASE1L3 protein expression levels in COAD.

### Immune infiltration analysis

The CIBERSORT algorithm was applied for obtaining the immune cell content of each colon cancer sample in the TCGA database for subsequent analysis. The correlation analysis between the expression of DNASE1L3 and immunocytes was performed by R language, and spearman rank correlation coefficient method was used for comparison.

### Tissue sample

The colon cancer tissue samples involved in this study were all from the Second Department of General Surgery, Guangdong Second Provincial General Hospital from August 2020 to December 2020. A total of 5 patients with colon cancer and adjacent normal tissue samples were collected. After surgery, all human tissue samples were quickly cut into tissue pieces of no more than 0.5 cm in width and height and placed in a cryopreservation tube with RNAlater solution in advance. The specimens are stored at 4° C at least overnight and then transferred to the -80° C refrigerator for long-term storage. All patients had not undergone interventional treatment or chemotherapy before surgery and had signed an informed consent form. The present study was approved by the Ethics Committee of Guangdong Second Provincial General Hospital.

### Cell lines

Human colon cancer cell lines: SW480, SW48, HT-29, HCT-116 and SW620 were donated by Professor Yang Xia from Sun Yat-Sen University. Cells were cultured in DMEM (Gibco, USA) containing 10% fetal bovine serum (Gibco, USA), 100 U/mL penicillin and streptomycin (Solarbio, GuangZhou, China), incubating at 37° C and 5% CO_2_ atmosphere. The DNASE1L3 overexpression plasmid and negative controls were designed and synthesized by Kidan Biosciences co., Ltd (Guangzhou, China). Transfection was performed using Attractene (Qiagen, USA) according to the manufacturer’s instructions.

### RNA extraction and quantitative real-time polymerase chain reaction (qRT-PCR)

The total RNA was extracted from colon cancer tissues using TRIzol reagent (AG, China). Then, cDNA was synthesized by reverse transcription according to Takara PrimeScript RTMaster Mix instructions. qRT-PCR was then performed in the light of SYBRGreen (TaKaRa, USA) instructions and detected by LC480 II. Relative gene expression was normalized to β-actin and calculated using the formula of 2^−ΔΔCt^. The primer sequences for DNASE1L3 were:5’-TGCTGCTTCTCCTCCTCTCCATC-3’(Forward) and 5’-TGCTGCTTCTCCTCCTCTCCATC-3’(Reverse). The primer sequences for β-actin were: 5’-GCACTCTTCCAGCCTTCCTT-3’(Forward) and 5’-GTTGGCGTACAGGTCTTTGC-3’ (Reverse).

### Western blotting analysis

Total proteins of cells were lysed with 1×SDS buffer. The protein concentration was determined using a BCA protein concentration quantification kit (Beyotime, ShangHai, China) according to the manufacturer’s protocol. Equal quality of proteins of every group were separated with 10% SDS-PAGE, and transferred to the PVDF membrane, then blocked in 5% milk for 1hr at RT. Membranes were incubated overnight at 4° C, with the primary antibody DNASE1L3 (mouse monoclonal antibody, Proteintech, 35KD, 1:1000), Tublin (rabbit monoclonal antibody, Beyotime, 55KD, 1:1000). The secondary antibody peroxidase anti-Rabbit (Vector Laboratories, PI-1000,1:1000), Peroxidase anti-Mouse (Vector Laboratories, PI-2000,1:1000) was incubated at RT for 1hr. At last, ECL detection kit (Beyotime, ShangHai, China) was used to detect the protein bands.

### Wound healing analysis *in vitro* assay

When the cell density of 6-well Plate reached about 70-80%, cells were transfected with DNASE1L3+ plasmid or NC plasmid. After the cells were filled with six plates, we used a sterile 200μl pipette tip to scratch each group, washed cells debris with warmed PBS, and placed into a fresh serum-free medium. Next, we took pictures at 0 h 24h and 48 h after scratching.

### Cell counting kit-8 (CCK8) assay

CCK8 (Beyotime, ShangHai, China) assay was applied to investigate the cell proliferation. The transfected cells were collected and planted into the 96-well plates with a density of 4000 cells/well, then cultured for an additional 24, 48 and 72hrs. Later, CCK8 solution (10μL) was added and cells treated for 2h at 37° C. Subsequently, the absorbance of 450 nm was measured.

### Statistics

All the experimental data were processed using software SPSS version 23.0 (IBM, Armonk, NY, USA) and Graphpad Prism version 5.0. Independent samples t-test was used for the intergroup comparison. *p* <0.05 was considered statistically significant.

## Supplementary Material

Supplementary Table 1
