# Supplementary material for: Deoxyribonuclease 1-like 3 may be a potential prognostic biomarker associated with immune infiltration in colon cancer
Source: Aging (Albany NY). 2021 Jun 22;13(12):16513–26. doi: 10.18632/aging.203173 (PMC8266351; doi:10.18632/aging.203173)
Supplement: Supplementary Table 1 [file aging-13-203173-s001.pdf]

SUPPLEMENTARY TABLE

Supplementary Table 1. DNASE1L3 related immune cells.

Cell	p value	R
Macrophages M0	1.01E-09	-0.5
T cells CD4 memory resting	2.04E-09	0.49
B cells naive	1.46E-05	0.36
Plasma cells	0.000293	0.31
Neutrophils	0.000654	-0.29
Monocytes	0.001023	0.28
Dendritic cells activated	0.001068	0.28
Dendritic cells resting	0.002178	0.26
Mast cells resting	0.03423	0.18
T cells regulatory (Tregs)	0.037038	-0.18
